# Systemic delivery of tenofovir alafenamide using dissolving and implantable microneedle patches

**DOI:** 10.1016/j.mtbio.2022.100217

**Published:** 2022-02-11

**Authors:** Alejandro J. Paredes, Fabiana Volpe-Zanutto, Lalitkumar K. Vora, Ismaiel A. Tekko, Andi Dian Permana, Camila J. Picco, Helen O. McCarthy, Ryan F. Donnelly

**Affiliations:** a School of Pharmacy, Queen's University Belfast, Medical Biology Centre, 97 Lisburn Road, Belfast, BT9 7BL, Northern Ireland, UK; b Department of Pharmaceutics, Faculty of Pharmacy, Hasanuddin University, Makassar, Indonesia

**Keywords:** Microneedles, HIV, Tenofovir alafenamide, Pre-exposure prophylaxis, PLGA

## Abstract

The human immunodeficiency virus (HIV) remains a global health concern, with 37.7 million people currently living with the infection and 1.5 million new cases every year. Current antiretroviral (ARV) therapies are administered through the oral route daily, often in lifelong treatments, leading to pill fatigue and poor treatment adherence. Therefore, the development of novel formulations for the administration ARV drugs using alternative routes is actively sought out. In this sense, microneedle array patches (MAPs) offer a unique user-centric platform that can be painlessly self-applied to the skin and deliver drugs to the systemic circulation. In this work, dissolving and implantable MAPs loaded with the tenofovir alafenamide (TAF) were developed with the aim of releasing the drug systemically. Both MAPs were sufficiently strong to pierce excised neonatal full-thickness porcine skin and form drug depots. *In vitro* release experiments performed in dialysis membrane models, demonstrated a relatively fast delivery of the drug in all cases. Franz cells experiments revealed that dissolving and implantable MAPs deposited 47.87 ​± ​16.33 ​μg and 1208.04 ​± ​417.9 ​μg of TAF in the skin after 24 ​h. Pharmacokinetic experiments in rats demonstrated a fast metabolization of TAF into tenofovir, with a rapid elimination of the metabolite from the plasma. The MAPs described in this work could be used as an alternative to current oral treatments for HIV management.

## Highlights

• HIV is still a global concern, with 38 million people currently living with the infection.
• ARV therapy can prevent HIV spread and extend life expectancy in infected people.
• MAPs constitute an attractive platform for the delivery of antiretroviral drugs including TAF.
• Dissolving and implantable TAF MAPs were obtained and characterised *in vitro* and *ex vivo*.
• Both MAPs were able to deliver TAF systemically in rats.

## Graphical abstract

**Figure:**
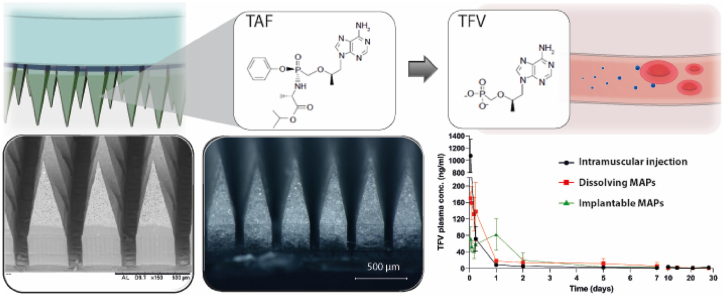

## 1 Introduction

The human immunodeficiency virus (HIV) remains a relevant health concern at a global level. With an estimate of 1.5 million new infections registered every year and 37.7 million people living with HIV in 2021, HIV has already claimed 33 million lives [1]. Even though there is a declining trend in the number of new infections, the 90% diagnosed, 90% on treatment and 90% virally suppressed target set by the Joint United Nations Programme on HIV and acquired immunodeficiency syndrome (UNAIDS) aimed by 2020 is still not being achieved [2]. One of the key factors in the fight against HIV is securing people access to antiretroviral (ARV) therapy, which in developed countries has been proven to improve people's life expectancy practically to the same level as the HIV-negative population. Nevertheless, in sub-Saharan Africa, the acquired immunodeficiency syndrome (AIDS, a progression from HIV infection) is still the leading cause of mortality amongst people in the 15–49 years age range, with women in particular, bearing the majority of positive cases in the HIV epidemic [3]. By June 2021, around 28.2 million people were receiving ARV drugs, constituting approximately 74% of adults and 54% of children infected with the virus [1].

ARV drugs (ARVs) are also used in HIV prevention. Pre-exposure prophylaxis (PrEP) *via* oral or topical vaginal and rectal administration of ARVs to non-infected people in vulnerable populations showed promising results in terms of HIV-1 infection prevention, however, clinical outcomes have varied extensively [[4], [5]]. This heterogeneous response to ARVs has been attributed to multiple factors, such as immunological status of patients, inter-subject pharmacokinetic variability and characteristics of the virus [6]. Currently available formulations require administration on a daily basis, often during lifelong periods, and suboptimal treatment adherence results in low and variable exposure, exacerbating risk of prevention or therapeutic failure [7]. Moreover, because most of the available regimens are oral, users may encounter pill fatigue, and this could potentially lead to suboptimal adherence, which has been identified as one of the leading causes of therapeutic failure and low rates of PrEP protection [8]. Furthermore, the oral route is often associated with erratic absorption, risk of chemical and enzymatic degradation, liver first pass metabolization, inter-subject variability, gastrointestinal side effects and restricted use in children and elderly patients [9]. Other formulations for ARV delivery include topical vaginal gels [10,11], vaginal rings [12], subdermal implants [13], and intramuscular (IM) long-acting injectables based on nanosuspensions [14]. Although the potential for these new preventive regimens is high, there are still considerations that need to be taken into account, such as training end-users on how to apply these products, discretion and users’ adherence in the long term. In particular for injectable formulations, the necessity of trained healthcare personnel and specific infrastructure, together with the creation of sharps waste, the possibility of accidental needle-stick injuries, needle re-use and needle-phobia might pose other challenges for a successful introduction and deployment of these formulations, especially in low- and middle-income countries. In this scenario, the use of microneedle array patches (MAPs) might be a useful strategy to circumvent these disadvantages and become an interesting alternative to deliver ARVs for both HIV treatment or prevention [15].

MAPs are minimally invasive devices which contain regularly spaced micro-sized projections (microneedles, MNs) that protrude from a baseplate and can by-pass the *stratum corneum* depositing their drug load in the viable layers of the skin [16]. Dissolving/bioresorbable MAPs are fabricated using polymers that dissolve or gradually degrade upon contact with the fluids of the dermis, leading to drug dissolution and posterior diffusion to deeper layers where the skin blood micro-vessels can pick the active up and distribute it to the body by means of the systemic circulation [16,17]. Furthermore, due to the short length of the MNs, MAPs can be self-applied without producing bleeding or pain, thus enhancing user compliance and treatment adherence [18]. Moreover, because dissolving MAPs do not produce sharps waste, needle-stick injuries and needle reuse can be avoided, and no specific disposal infrastructure is required. Crucially, because MAPs are water-free and do not require cold chain storage, they could be easily distributed to distant non-urban areas [19]. All these advantages make of MAPs an attractive alternative to currently available ARV regimens.

Tenofovir alafenamide (TAF) is a prodrug of the nucleotide homologue tenofovir (TFV) and is co-administered with other drugs for the management of both HIV and hepatitis B, and for HIV PrEP [20,21]. In comparison to the previous TFV prodrug, tenofovir disoproxil fumarate (TDF), TAF presents much safer renal and bone profiles, since it has an increased stability in plasma than TDF, which produces lower levels of TFV in the systemic circulation and preferential distribution and accumulation of the drug in the lymphatic cells and tissues [22]. Several studies described the *in vitro* and *in vivo* delivery of TAF from implants for weeks or months [[23], [24], [25], [26], [27]]. However, these systems need a surgical procedure to be implanted and removed, which represents a major challenge, especially in sub-Saharan Africa where HIV is more prevalent and healthcare infrastructure is limited or even inexistent. Another report described a transdermal patch that could deliver the drug for a week in rats, but a large area of the skin must be covered by the patch, which must also remain in place during the treatment [28]. In this context, the development of a microneedle-based formulation that can be self-applied and deliver the drug systemically after a short-duration, single application is an interesting proposition.

**Fig. 1:**
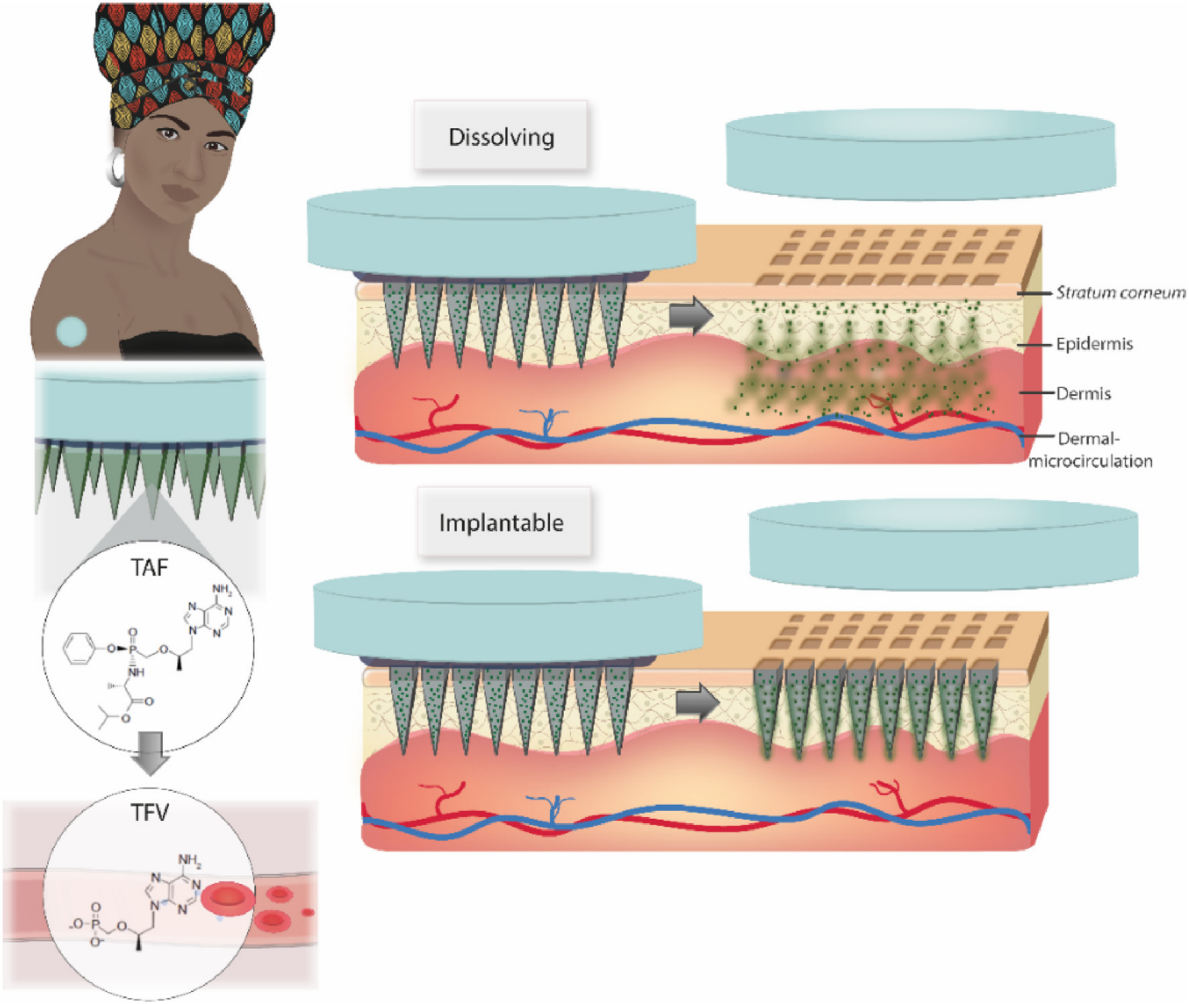
Intradermal administration of TAF using dissolving and implantable PLGA MAPs.

Here, we describe, for the first time, the administration of TAF using MAPs as illustrated in Fig. 1. Dissolving and implantable formulations were manufactured by a micromoulding methodology and characterised in terms of mechanical properties. The *in vitro* release profile of TAF from the dissolving and implantable matrices was evaluated using dialysis membranes. Ex vivo skin experiments in Franz cells were used to study the skin deposition and transdermal delivery of the novel MAPs. Finally, the pharmacokinetic performance of the novel MAPs was studied in rats.

## 2 Materials and methodology

### 2.1 Materials

TAF (also known as GS-7340) was provided by Gilead Sciences, Inc. TAF and TFV and their stable isotopically labelled (SIL) internal standards (TFV-d6 and TAF-d5) were obtained from Toronto Research Chemicals (Canada). Poly(vinylpyrrolidone) (PVP K29-32; and PVP K90) was provided by Ashland (Kidderminster, UK). Poly(vinyl alcohol) (PVA 9–10 ​kDa) and solvents methanol ≥99.9%, dimethyl sulfoxide (DMSO), tetrahydrofuran (THF) inhibitor-free ≥ 99.9% and acetonitrile (ACN) ​≥ ​99.9% (all HPLC grade), ammonium acetate ≥98%, and Acetic acid ≥99.7% were purchased from Sigma-Aldrich, St. Louis, USA. Two different types of poly(d,l-lactide-co-glycolide) (PLGA) were used, a low viscosity PLGA, Purasorb® PDLG 7502 with a lactide:glycolide ratio of 75:25 and ester end (Purac®, Corbion, Netherlands) (LV-PLGA) and a high viscosity PLGA with a lactide:glycolide ratio of 75:25 and ester end (Viatel® DLG 7503 ​E) (HV-PLGA). Both PLGAs were a donation from Ashland (Kidderminster, UK). All other reagents used in this work were of analytical grade and purchased from Sigma-Aldrich. Elga purified water was used in all cases (Purelab option® Elga LabWater, High Wycombe, UK).

### 2.2 MAPs manufacture

**Fig. 2:**
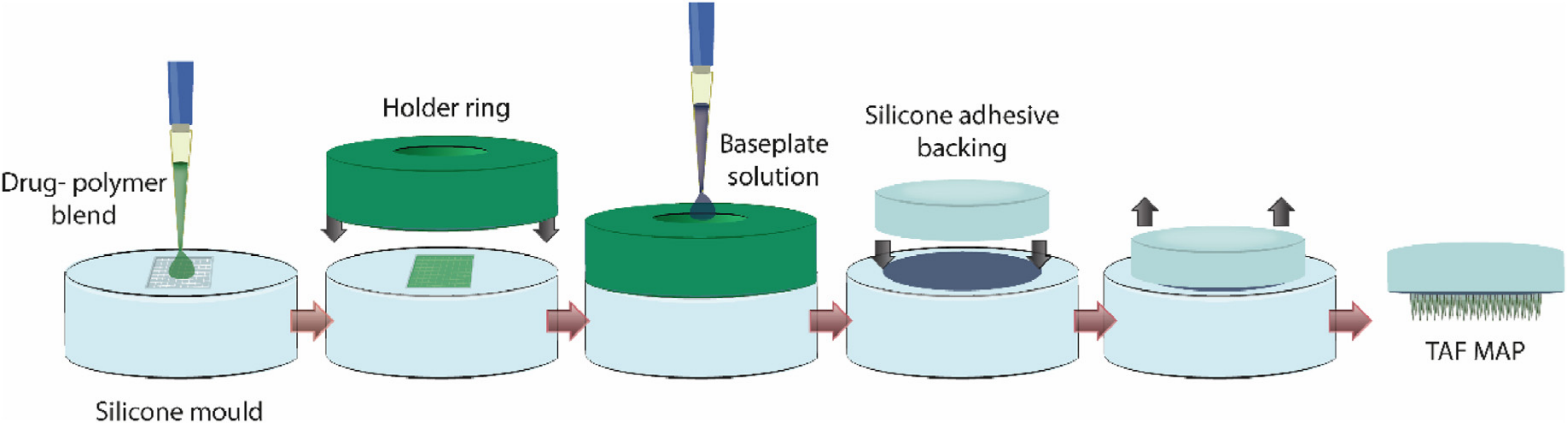
Illustration of the TAF MAPs formulation protocol.

MAPs were prepared using silicone moulds with pyramidal geometry (600 ​μm pyramidal tip, 250 ​μm cuboidal base column), 300 ​μm width at the base, 300 ​μm interspacing between MNs, needle height 850 ​μm, and a needle density of 16 ​× ​16 (on 0.5 ​cm^2^). For the dissolving MAPs, the first layer containing the drug was prepared from a blend consisting of 0.84 ​g of TAF, 1.2 ​g 20% w/w of PVA (9–10 ​kDa) and 20% w/w PVP K29-32 (1:1), and 1 ​ml of water. All the components were homogenized at 5000 ​rpm for 10 ​min using a SpeedMixer™ (DAC 150 FVZ, High Wycombe, England). The implantable MN tips made of two different types of MAPs obtained from LV PLGA and HV PLGA. To this purpose, 0.3 ​g of PLGA were dissolved in 500 ​μl of DMSO with the same SpeedMixer™ (2 cycles of 3 ​min at 3000 ​rpm), then 0.3 ​g of the drug were gradually added in three parts by homogenising each time for 1 ​min with the SpeedMixer™ at 3000 ​rpm. An excess of the resultant blends was poured onto the silicone moulds and placed in a positive pressure chamber at 5 ​bar for 2.5 ​min. The excess formulation was then removed from the top of the moulds using a spatula, and a silicone ring insert with an internal diameter of 18 ​mm, external diameter of 23 ​mm and thickness of 3 ​mm was attached to the silicone moulds using as a glue a solution of PVA (9–10 ​kDa) 40% w/w. Dissolving tips were dried for 4 ​h at room temperatures and PLGA formulations for 24 ​h on the bench, plus a further 24 ​h in a desiccator under vacuum to ensure the removal of the DMSO. Once the first layers were dried, 150 ​μl of 30% w/w PVP K90 were added using a positive displacement pipette, and the systems were centrifuged for 6 ​min at 4332 ​*g*. The systems were left overnight for evaporation of the water in the second layer and the MAPs were separated from the moulds using a transparent silicone adhesive wafer of 10.91 ​mm in diameter and 3.6 ​mm in height (3 ​M, USA). To ensure complete drying, MAPs were stored in an oven at 37 ​°C overnight. The simplified formulation protocol is represented in Fig. 2. A visual examination of the TAF MAPs was done using a stereo microscope (Leica EZ4W, Leica Microsystems, Milton Keynes, UK), a digital microscope (VHX, Keyence, Ltd, Milton Keynes, UK), and a scanning electron microscope (Tabletop TM 3030, Hitachi, Tokyo, Japan).

### 2.3 Mechanical characterization and insertion capacity

The mechanical properties of the MN tips were investigated using a texture analyser in compression mode (TA.XT2, Stable Micro Systems., Ltd., Haslemere, UK). A force of 32 ​N for 30 ​s was applied in vertical direction at a downward speed of 1.19 ​mm/s, which represents the average human force applied when applying MAPs, as demonstrated in previous studies [29,30]. MAPs were compressed against a flat stainless-steel surface and the height of the MNs was determined before and after the experiment using a stereomicroscope. The percentage of MNs height reduction was informed (*n* ​= ​6). Insertion studies were performed using 8 layers of Parafilm® as a simulant model for the skin. To this purpose, eight layers of Parafilm® were cut and stacked on top of each other. A good correlation between this model and skin was observed in previous studies [29]. Using the same texture analyser set-up, the insertion was measured in terms of the wholes created by the MAPs in each separated layer, which were counted with the help of a stereo microscope equipped with two polarizer filters (Leica EZ4W, Leica Microsystems, Milton Keynes, UK).

### 2.4 TAF recovery from whole MAPs, baseplates, and MN tips

Aiming to evaluate TAF content and distribution in the MAPs, MNs were carefully scraped from the baseplate with a scalpel. Entire MAPs, baseplates and MNs were individually placed in 5 ​ml of deionized water and stirred for 30 ​min using a magnetic stirrer, then 5 ​ml of methanol were added, and the system agitated for a further 30 ​min. PLGA MNs were sonicated for 1 ​h in the water-methanol solution to ensure complete dissolution of the drug. An aliquot of the solution was taken and diluted 1:1 v/v with ACN to precipitate polymers, centrifuged at 14,462 ​*g* (Sigma microtube centrifuge SciQuip Ltd, Shropshire, UK) for 15 ​min, diluted if required, and quantified by HPLC.

### 2.5 Fourier transform infrared spectroscopy (FTIR)

Infrared spectroscopic patterns of the pure drug, dissolving and implantable formulations were obtained. Analyses were performed using an Accutrac FTIR (FT/IR-4100 Series, Jasco, Essex, UK) equipped with Diamond MIRacle™ ATR, in the region of 4000–600 ​cm^−1^ ​at a resolution of 4.0 ​cm^−1^. An average of 64 repeat scans were taken to obtain each spectra.

### 2.6 Thermal analysis

Differential scanning calorimetry (DSC) and thermogravimetric analysis (TGA) were performed to the pure drug, dissolving and implantable formulations. DSC analyses were performed using a TA Instruments DSC (Q100, TA Instruments, New Castle, DE, USA). The temperature range was set from 30 to 200 ​°C, the heating speed was fixed at 10 ​°C/min and the nitrogen flow rate was 10 ​ml/min. TGA was carried out using a thermogravimetric analyser (Q500, TA Instruments, New Castle, DE, USA). The heating rate was set at 10 ​°C/min in the range of 25–450 ​°C.

### 2.7 Powder X-ray diffraction

The crystallinity of TAF in pure state and included in MAPs was evaluated using a powder diffractometer (Miniflex X-ray, Rigaku Corporation, Kent, England) with Cu Kα radiation, between 5° and 60° in 2θ in steps of 0.04 and fixing the counting time at 0.5 ​s per step.

### 2.8 Drug stability

The stability of TAF was evaluated in PBS, water, buffer ammonium acetate 30 ​mM (pH 6.0), methanol, water with 0.2% v/v of formic acid (pH 4.18), and PBS with 0.2% v/v of formic acid (pH 6.65). Solutions of TAF (10 ​μg/ml) were prepared and stored at room temperature, the amount of drug was quantified by HPLC at predetermined time intervals over 24 ​h. Each solution was prepared and assayed in triplicate (*n ​=* ​3).

### 2.9 *In vitro* release studies

The *in vitro* release profiles of TAF and TAF-MN powder were evaluate using a dialysis membrane model using a previously described methodology [31,32]. Briefly, 10 ​mg of TAF and an amount of TAF-MN powder equivalent to 10 ​mg of the drug were dispersed in 1000 ​μl of PBS and placed into dialysis membrane bags (Spectra-Por®, 12,000–14,000 MWCO, Spectrum Medical Industries, Los Angeles, CA, USA) and closed using plastic clamps. As release media, 100 ​ml of acetate buffer (30 ​mM, pH 6.0) were used. The hermetically sealed containers were placed in a ISF 7100 orbital incubator with an agitation speed of 100 ​rpm and a temperature of 37 ​°C (Jeio Tech, Ma, USA). 1000 ​μl-samples were withdrawn at predetermined timepoints until 48 ​h and replaced with fresh buffer and quantified using the HPLC method described in the following Section. The experiments described in this Section were performed in quadruplicate (*n ​=* ​4).

### 2.10 TAF quantification

The concentrations of TAF in each study were determined by HPLC (Agilent Technologies 1220 Infinity UK Ltd, Stockport, UK). The column used was a SphereClone™ C_18_ ODS (pore size, 80 ​Å; length, 150 ​mm; internal diameter, 4.6 ​mm; particle size, 5 ​μm) (Phenomenex, Torrance, CA, USA). The temperature of the column was fixed at 25 ​°C. A successful chromatographic TAF separation was achieved using a mobile phase composed of 65% ammonium acetate buffer 0.03 ​M (adjusted to pH 6.0 with acetic acid) and 35% of THF:ACN (70:30% v/v). The maxima absorption (*λ*_max_) was fixed at 260 ​nm, the injection volume was 20 ​μl, and the flow rate was 1 ​ml/min.

### 2.11 Skin deposition and transdermal cumulative permeation in Franz cells

*Ex vivo* skin deposition studies of MAPs containing TAF were performed in excised neonatal full-thickness porcine skin. To this purpose, the skin was carefully shaved and attached to the donor section of the Franz cells using cyanoacrylate glue. TAF formulations were applied to the skin using manual force for 30 ​s. Then, the donor compartment was joined to the receptor chamber containing 12 ​ml of ammonium acetate buffer (30 ​mM, pH 6.0). Afterward, a 10 ​g cylindrical weight made of stainless-steel was placed on top of each TAF MAP. The sampling arm and the donor compartment were closed using Parafilm® to avoid the evaporation of the release medium. The temperature of the system was maintained at 37 ​± ​1 ​°C using a water circulator (Julabo Corio C, Cole Palmer, Vernon Hills, Illinois, USA). Magnetic agitation of the release medium was kept constant at 600 ​rpm during the experiment. After 24 ​h, TAF MAPs were detached, and the skin was rinsed twice with 500 ​μl of PBS in order to remove any excess formulation. Subsequently, a 10 ​mm-diameter biopsy punch was used to obtain a full-thickness section of skin from which the drug was extracted. To this purpose, each piece of skin was placed in a 2 ​ml Eppendorf tube, frozen and cut in small pieces using scissors. Afterward, three stainless steel beads (0.5 ​cm diameter, Qiagen, Hilden, Germany) and 1.5 ​ml of methanol 70% (v/v) previously stored at 5 ​°C were added. The tubes were then placed in a TissueLyser® LT (Qiagen, Hilden, Germany) and processed for 15 ​min at 50 ​Hz. The resultant homogenates were immediately centrifuged at 14,462 ​*g* (Sigma microtube centrifuge SciQuip Ltd, Shropshire, UK) for 15 ​min and the supernatant filtered and suitable diluted for TAF quantification by HPLC. The TAF concentration in the receptor compartment after 24 ​h was assayed by HPLC. Using the same experimental set-up, the transdermal cumulative release of TAF from dissolving and implantable MAPs was evaluated by taking 0.25 ​ml samples from the receptor compartment with replacement of fresh medium at predetermined time intervals. After filtration and suitable dilutions, TAF was quantified by HPLC. The delivery efficiency of the formulations was calculated using Equation (1).

(1) $$Delivery\text{ ​}efficiency\text{ ​}\left(\%\right)\text{ ​}=\text{ ​}\frac{TAF_{skin}+\;TAF_{Receptor}}{TAF_{MAP}}\;x\;100\%$$

where TAF_skin_ refers to the amount of TAF extracted from the skin, TAF_receptor_ is the amount of drug quantified in the receptor compartment of the Franz cells and TAF_MAP_ is the drug content in the whole MAP. The experiments reported in this section were carried out by sextuplicate (*n ​=* ​6).

#### 2.11.1 Optical coherence tomography

The skin structure and the insertion of TAF MAPs was assessed by optical coherence tomography (OCT). To this purpose, the skin samples collected after the skin deposition experiments were gently cleaned to remove any superficial gel residue and analysed using an OCT microscope (EX1301, Michelson Diagnostics Ltd., Kent, UK). Optical microscopy images were also taken as a complement of OCT analyses.

### 2.12 Pharmacokinetics

Pharmacokinetic studies were carried out in female Sprague Dawley rats aged between 9 and 13 weeks at the starting point of the study. The animals could access water and food *ad libitum* throughout the duration of the experiment. Three groups of six rats (*n ​=* ​6) were treated with dissolving MAPs, implantable MAPs and an IM injection of 4 ​mg of TAF in 50 ​μl of a freshly prepared aqueous suspension (control group), respectively. In the groups treated with MAPs, a total of 4 patches were applied to each animal and blood samples were withdrawn from the tail vein at defined time intervals up to 4 weeks [33,34]. The *in vivo* experiments were carried out with ethical permission from the Queen's University Belfast, Biological Services Unit (BSU). All the researchers involved in the study held Personal Licences from the UK Home Office. Plasma samples of 150 ​μl were obtained by centrifugation of the blood at 1000 ​*g* and 4 ​°C for 10 ​min, then 12 ​μl of 20% v/v formic acid were added to preserve TAF stability and stored at −20 ​°C for extraction and HPLC quantification.

#### 2.12.1 TAF quantification in plasma samples

TAF and TFV concentrations in plasma samples were determined using a previously validated LC-MS/MS method [35]. The chromatographic system consisted of an ExionLC™ AD pump multiplate sampler and pump (ABSciex Limited, Cheshire, United Kingdom). Chromatographic separation was possible using a Synergi RP-C18 Phenomenex® column (3 ​μm, 15 ​cm ​× ​2.1 ​mm) and a gradient elution method with the flow rate fixed at 0.5 ​ml/min. The mobile phase gradient started with 98% mobile phase A [0.1% (v/v) formic acid in deionized water], which was held for 0.2 ​min then increasing in organic content to 30% mobile phase B [0.1% (v/v) formic acid in acetonitrile] which was maintained up to 2.5 ​min followed by a return to aqueous conditions (98% mobile phase A) in order to recondition the column over a total run time of 6 ​min. Detection and quantification of TAF and TFV were achieved with an 4500 Triple Quadrupole from ABSciex Limited (Cheshire, UK) with an electrospray ionization source, the capillary voltage at 4.0 kV/300 ​°C, vaporizer at 350 ​°C, and the collision gas flow (Ar) 1.5 mTorr. Tuning and data acquisition were carried out using Analyst 1.7 and processing/quantification using MultiQuant 3.0.3 (AB Sciex Limited). The triple quadrupole mass spectrometer was operated in positive ion mode using selective reaction monitoring (SRM). The *m/z* transitions were, TFV (288.1 ​→ ​176.2), TAF (477.2 ​→ ​176.2) and labelled SILs TFV-d6 (294.1 ​→ ​182.1) TAF-d5 (482.3 ​→ ​176.1). The assay calibration curve ranges were from 1 to 1000 ​ng/ml (TFV) and 0.5–500 ​ng/ml (TAF), respectively.

Calibrators were prepared fresh on the day of analysis using acidified rat plasma (20% v/v formic acid), containing 100 ​μl of each calibrator (in duplicate) and 20 ​μl of deuterated internal standard (TFV-d6 and TAF-d5; 400 ​ng/ml). Analytes were extracted from plasma samples using SPE cartridges (strong cation exchange cartridge; Thermo Fisher Scientific). SPE columns were conditioned with methanol followed by distilled water (500 ​μl). The standards, QCs and rat samples (in 1% v/v formic acid) were loaded onto the SPE cartridges and centrifuged for 2 ​min at 1000 ​rpm. Analytes were eluted with 500 ​μl of 1% (v/v) formic acid in water followed by 500 ​μl of 1% (v/v) formic acid in methanol and the cartridges were centrifuged for 2 ​min at 1000 ​rpm. The eluent was evaporated under nitrogen at room temperature until dry and reconstituted with 100 ​μl (99:1, v/v) water:ACN. The reconstituted samples were vortexed (∼5 ​s), transferred to vials, and analysed by using LC-MS/MS instrument. The validation data corresponding to the bioanalytical method used in this work is presented in Table 1.

**Table 1:** Validation data of the bioanalytical HPLC method.

Analyte	TAF	TFV
Retention time	3.0 ​min	1.4 ​min
LLQ	0.5 ​ng/ml	1.0 ​ng/ml
ULQ	500 ​ng/ml	1000 ​ng/ml
Intra-assay (%CV)	9.14 (LQC), 8.38 (MQC), 3.35 (HQC)	12.4 (LQC), 8.48 (MQC), 3.74 (HQC)
Inter-assay (%CV)	6.60 (LQC), 6.97 (MQC), 2.90 (HQC)	9.11 (LQC), 6.68 (MQC), 3.36 (HQC)

LLQ, Lower limit of quantification; ULQ, Under limit of quantification; LQC, Low quality control; Medium quality control; HQC, High Quality Control. %CV ​= ​% coefficient of variation.

#### 2.12.2 Calculation of pharmacokinetic parameters

The pharmacokinetic profiles of the IM control and microneedle formulations were constructed by plotting TFV plasma concentrations against time and analysed using a non-compartmental model. The pharmacokinetic parameters mean residence time (MRT), area under the curve (AUC), time of maximum concentration (T_max_), maximum drug concentration (C_max_) and mean half-life (t_1/2_) were calculated using PK solver software [36]. The relative plasma bioavailability (F) of TAF following MAP administration considering the IM injection as a comparator was estimated using Equation (2).

(2) $$\text{F ​}=\text{ ​}\frac{AUC_{MAP}\;x\;dose_{IM}}{AUC_{IM}\;x\;dose_{MAP}}\;x\;100\%$$

where, AUC_MAP_ is the AUC value obtained after the respective MAP patch application, AUC_IM_ is the AUC after IM injection of TAF. The dose for the IM injection was 4 ​mg, whereas for the MAPs, the dose was considered as the amount of TAF loaded in the MN tips.

### 2.13 Statistical analysis

Statistical analysis was performed using the GraphPad Prism© software (version 8.0, GraphPad Software Inc, San Diego, California, USA). An unpaired *t*-test was applied to analyse the results from two cohorts. To compare more than two cohorts, one-way ANOVA was applied. All the results are expressed as means ​± ​SD. In all cases, a *p* value ​< ​0.05 denoted significance.

## 3 Results and discussion

### 3.1 Manufacture of TAF MAPs

**Fig. 3:**
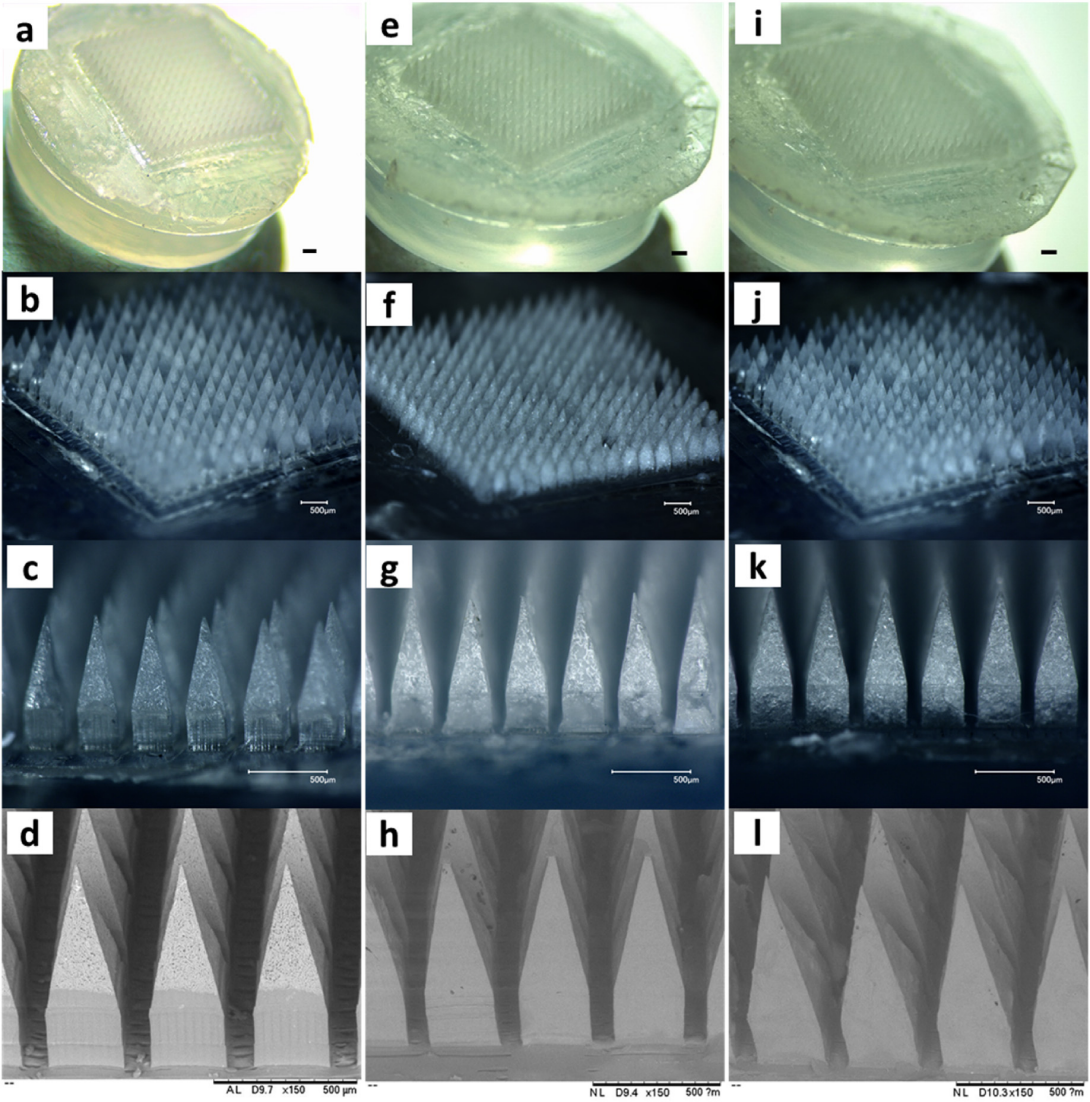
Microscopical analyses of TAF-loaded dissolving and implantable MAPs. **a-c-** Optical microscopy of dissolving MAPs, **d-** Scanning electron microscopy of dissolving MAPs. **e-g-** Optical microscopy of implantable LV-PLGA MAPs, **h-** Scanning electron microscopy of LV-PLGA. **i-k-** Optical microscopy of implantable HV-PLGA MAPs, **l-** Scanning electron microscopy of HV-PLGA. The scale bar is, in all cases 500 ​μm.

TAF dissolving MAPs were successfully obtained using an aqueous blend of PVP 20% w/w and PVA 20% w/w in a mass ratio of 1:1. The use of these two excipients has been demonstrated to be beneficial for the mechanical properties of the final formulation when compared to the polymers used individually, and this is related to the interaction between the C=O groups of PVP and the OH groups of PVA [37]. As shown in Fig. 3a, MNs were fully formed and all of them were attached to the transparent silicone wafer. Moreover, the electronic microscopy of this formulation evidenced the presence of two clearly distinguished phases with TAF in the form of needle-shaped crystalline particles being concentrated in the MN tips (Fig. 3b). In the case of the MAPs prepared with LV- and HV-PLGA, both formulations also presented a high uniformity, and SEM photomicrographs evidenced a smooth surface in both systems (Fig. 3c–f). Owing to the hydrophilic nature of TAF and its short half-life [22], the use of PLGA as matrix material in the MNs may allow to slower the release rate of TAF *in vitro* and in *vivo*. PLGA is an attractive polymer for the formulation of implantable MAPs, as it have the potential to entrap the active and release it in a sustained manner [38]. Both dissolving and implantable MAPs presented MNs attached to the baseplate, which was facilitated by the addition of a linker polymer (PVP K90) that enable the removal of the patches once the formulations were dried. This simple and flexible microfabrication method permitted a rapid casting of the first and second layers, and an easy removal of the patches from the moulds using the adhesive silicone wafers, obtaining highly uniform, bubble-free, and easily manoeuvrable formulations.

### 3.2 Mechanical characterisation

**Fig. 4:**
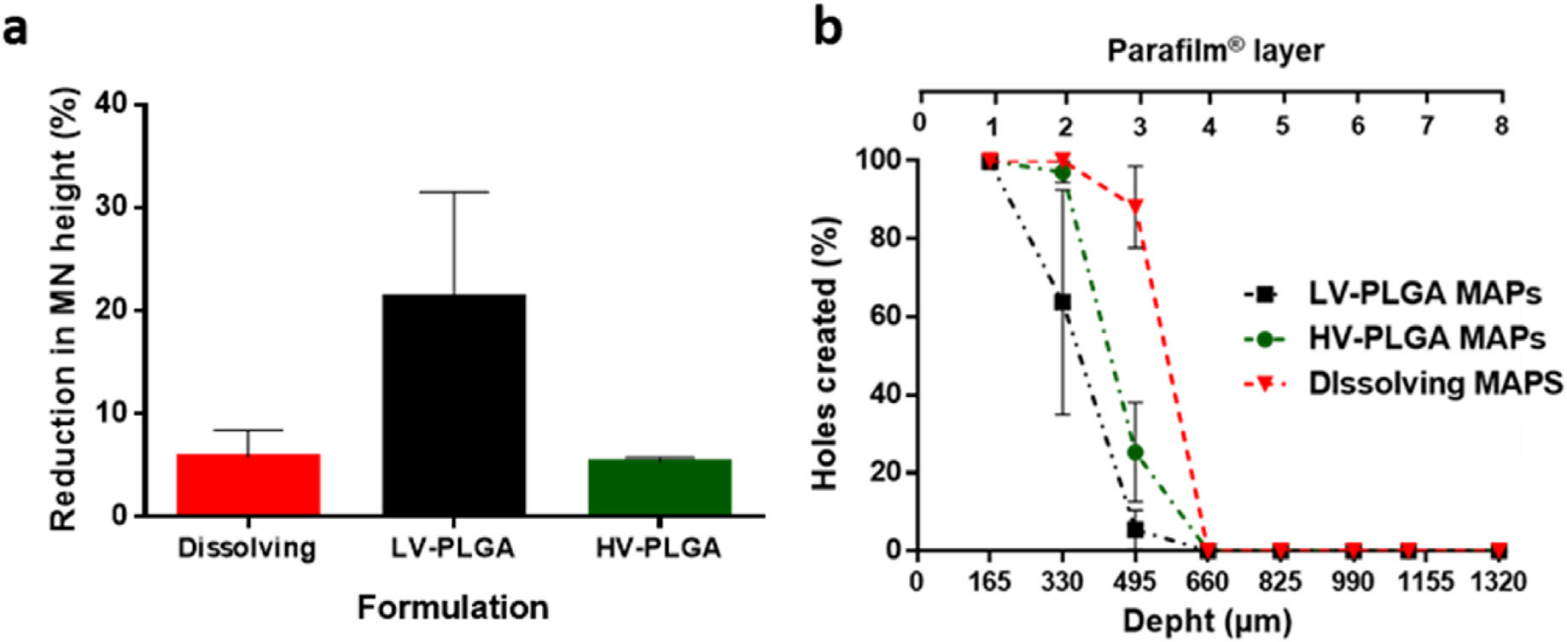
Mechanical characterisation of dissolving and implantable MAPs. **a-** MN height reduction and **b-** MN insertion capacity in the skin simulant Parafilm® model.

Crucial information regarding the mechanical properties of MAPs can be obtained by evaluating MNs height reduction after a compression force is applied. As shown in Fig. 4a, dissolving MN tips presented a height reduction of 5.83 ​± ​2.54%, which is in agreement with the data observed in previous works where similar polymer compositions and MN geometry were used in the manufacturing process [32,39]. When LV-PLGA was used as main component of the MN tips, poor mechanical resistance was observed, and the MN height was reduced in average by 23.29%, with a high degree of intra-batch variation. On the other hand, HV-PLGA produced stronger MNs that reduced their height only by 5.83%, a similar value to that observed for the dissolving formulation. The height reduction trend observed for PLGA MN tips corresponds to the inherent viscosity of the polymers, being 0.2 ​dl/g for LV-PLGA and 1.0 ​dl/g for HV-PLGA. The Parafilm® skin-simulant insertion test presented in Fig. 4b showed that the dissolving TAF MAPs were able to pierce the first two layers of Parafilm® with nearly all the MNs, while the third layer was pierced by 88.15% of the MNs. LV-PLGA MAPs showed poor penetration capability, piercing the first layer with 100% of the MNs, and 63.77% in the second layer, whereas HV-PLGA were able to pierce the first and second Parafilm® layers with 99.9% and 97.1% of the MNs, respectively. MAPs able to pierce two or more Parafilm® layers (or 330 ​μm in depth) have the potential to deliver actives intradermally, localising the drug in the skin, but also leading to systemic distribution, as demonstrated in previous reports [34,40,41]. Although there is a lack of specific standardized quality controls for MAPs [42], the height reduction of the MN tips and their penetration capacity in the Parafilm® skin simulant model are two complementary experiments allow to predict the behaviour of the MAPs when inserted in the skin both *in vitro* and *in vivo.*

### 3.3 Stability of TAF

**Fig. 5:**
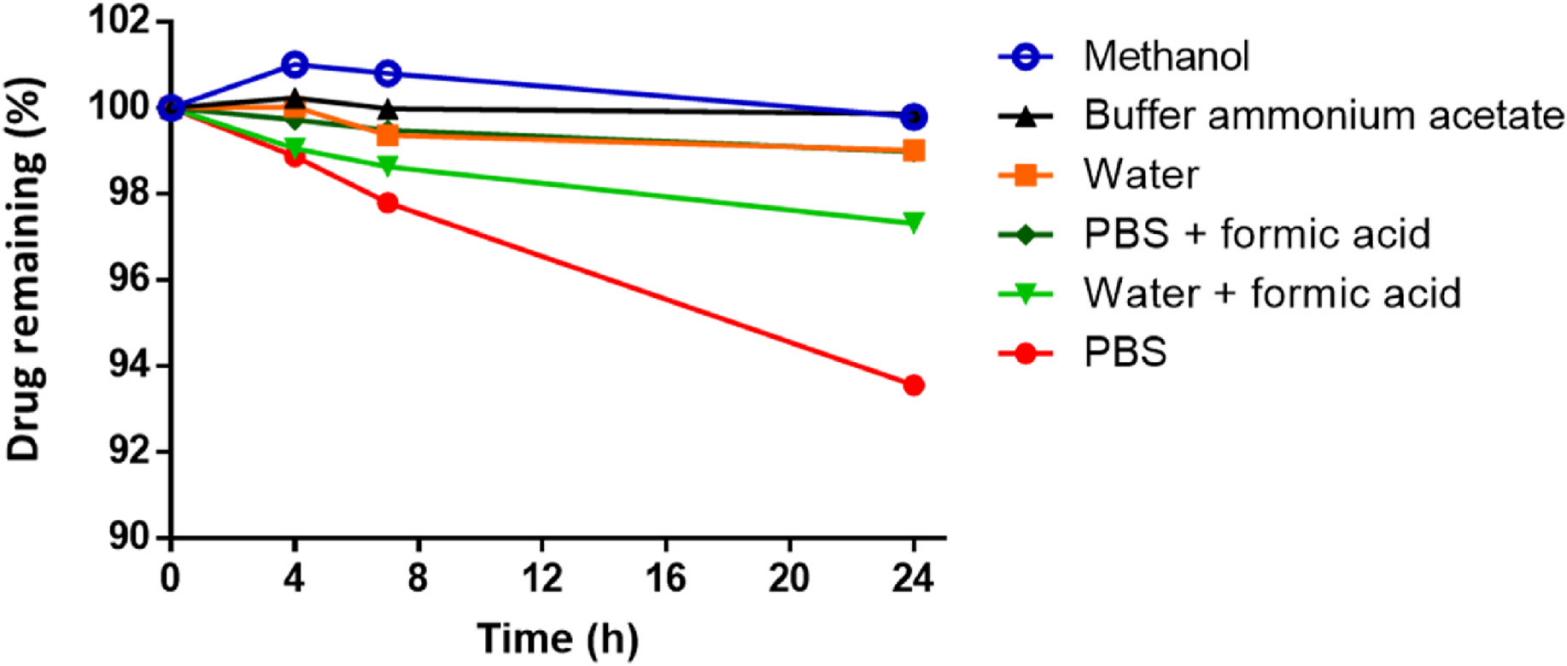
Chemical stability of TAF in different solutions in a 24 h-experiment (drug concentration: 10 ​μg/ml, room temperature). Experiments were performed in triplicate (*n* ​= ​3).

As observed in Fig. 5, TAF was found to be stable in ammonium acetate buffer and methanol, since 99.76% and 99.89% of the original concentration of the drug was quantified after 24 ​h, respectively. The stability of TAF in the other evaluated dissolution media expressed in descending order was: pure water and PBS with formic acid (99.01 and 98.99% respectively) ​> ​water with formic acid (97.32%) ​> ​PBS (93.56%). The stability TAF was evaluated in other reports, where strong basic pH and PBS were detrimental to the drug, with higher stabilities found at the pH range 5–6 [43]. Considering these results, the buffer ammonium acetate was used for further experiments.

### 3.4 Physicochemical characterisation

**Fig. 6:**
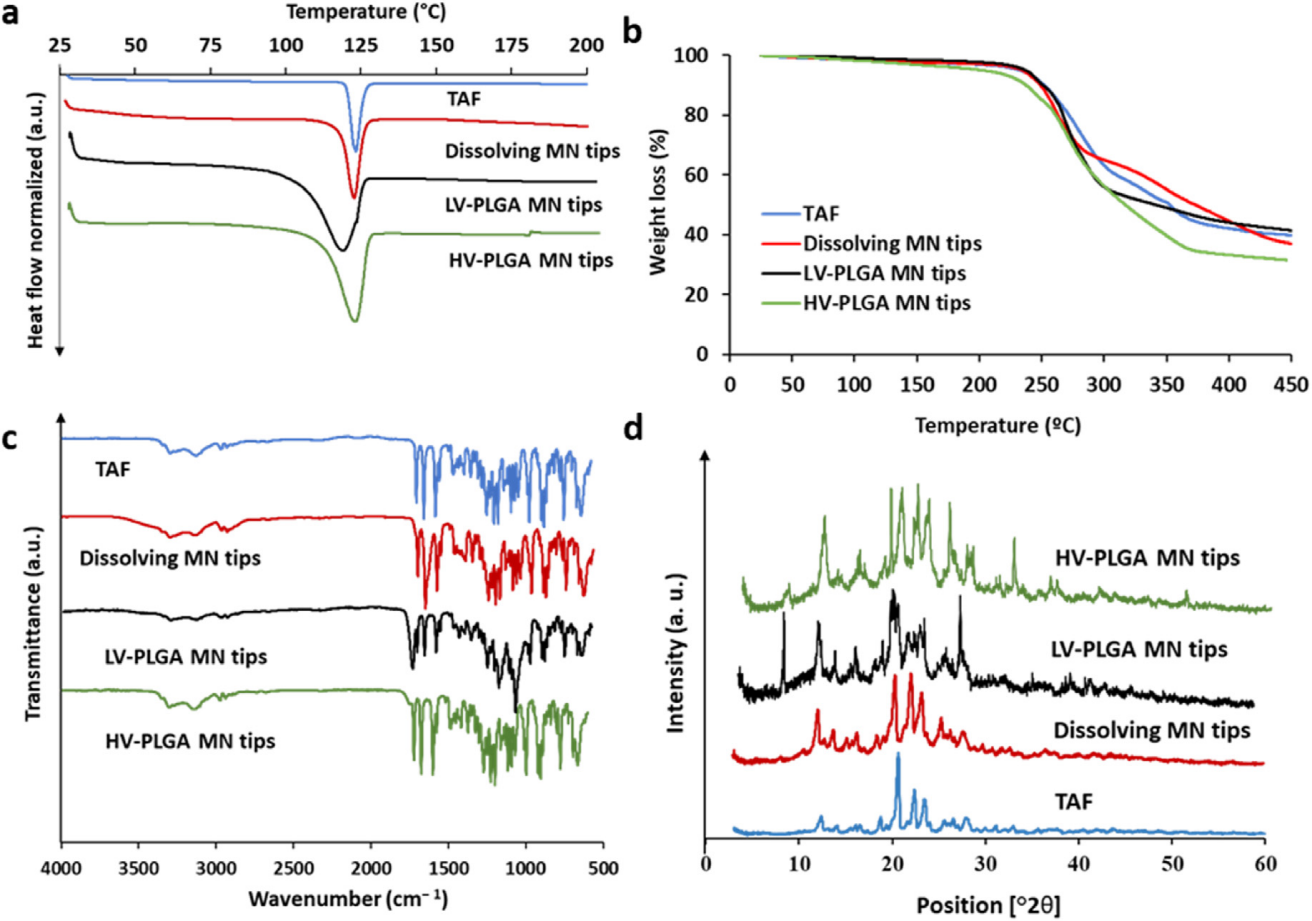
Physicochemical characterisation of TAF, dissolving, and LV-, HV-PLGA implantable MN tips. **a-** Differential scanning calorimetry (DSC). **b-**thermogravimetric analysis (TGA). **c-** Fourier transformed infrared spectroscopy (FTIR). **d-** Powder X-ray diffractometry.

Critical information about the crystalline characteristics of the drug and its interaction with other materials in the formulation are reported in this section. DSC experiments presented in Fig. 6a revealed that the characteristic endothermic melting peak of TAF (120.43 ​°C) was also present in the dissolving and implantable MAPs, indicating that TAF maintained its crystalline state after the formulation process. The peaks corresponding to LV- and HV-PLGA MN tips showed a broader range of melting temperatures, which can be attributed to the amorphous characteristics of PLGA [41]. The thermogravimetric profiles (Fig. 6b) were also similar for pure TAF and the formulations assayed, showing negligible weight losses until 200 ​°C, which indicates a correct solvent removal in the casting process. Between 200 and 450 ​°C, a decomposition of the samples with a weight loss of approximately 50% was observed. FTIR analysis was used to evaluate potential interactions between the TAF and the excipients used. The FTIR spectra of TAF alone and loaded into different MAPs indicated no interaction between TAF and the excipients, since all the characteristic stretching bands of the drug were also present in the formulations, as can be observed in Fig. 6c. For instance, the stretching bands detected for TAF at 1410 and 1450 ​cm^−1^ (aromatic C=N), 1600 ​cm^−1^ (N–H), 1673 ​cm^−1^ ​(P=O), 3137 ​cm^−1^ (aromatic C–H) and 3300 ​cm^−1^ (O–H) were also present in the dissolving and implantable MN tips. Finally, the powder X-ray diffraction patterns observed in Fig. 6d indicate that the drug remained crystalline after its incorporation into dissolving and implantable MAPs. The characteristic crystalline peaks of bulk TAF distinguishable at 12, 21, 23 and 24°2θ were also found in the MN formulations. In agreement with the data collected from DSC experiments, this confirms the crystalline characteristic of the drug both in its bulk state and when included in both types of MAPs.

### 3.5 Drug content

The drug content in whole MAPs, MN tips and baseplates is presented in Table 2. The three formulations evaluated were able to load similar amounts of TAF in the whole MAPs, the separated MN tips and baseplates. Expectedly, most of the drug was located in the tips, whereas some residual drug was quantified in the baseplates. Due to the fact that TAF is slightly soluble in water (5.63 ​± ​0.54 ​mg/ml), a back-migration of the active is produced when the second layer is added on top of the MNs. The formulation approach reported here, which involves the addition of a small volume of gel to produce the adhesion between the formed MNs and the dry pre-formed baseplate, reduces this phenomenon, leading to the concentration of nearly 85% of TAF in the MN tips.

**Table 2:** TAF content of dissolving and PLGA MAPs determined in whole MAPs, MN tips and baseplates.

	Formulation		
Sample	**Dissolving MAPs**	**LV-PLGA MAPs**	**HV-PLGA MAPs**
Whole MAPs (mg)	2.51 ​± ​0.14	2.61 ​± ​0.24	2.81 ​± ​0.42
MN tips (mg)	2.26 ​± ​0.17	2.20 ​± ​0.22	2.37 ​± ​0.27
Baseplate (mg)	0.27 ​± ​0.04	0.32 ​± ​0.06	0.33 ​± ​0.05

MAPs, microneedle array patches; LV-PLGA, Low viscosity poly lactic-co-glycolic acid; HV-PLGA, High viscosity poly lactic-co-glycolic acid.

### 3.6 *In vitro* release study

**Fig. 7:**
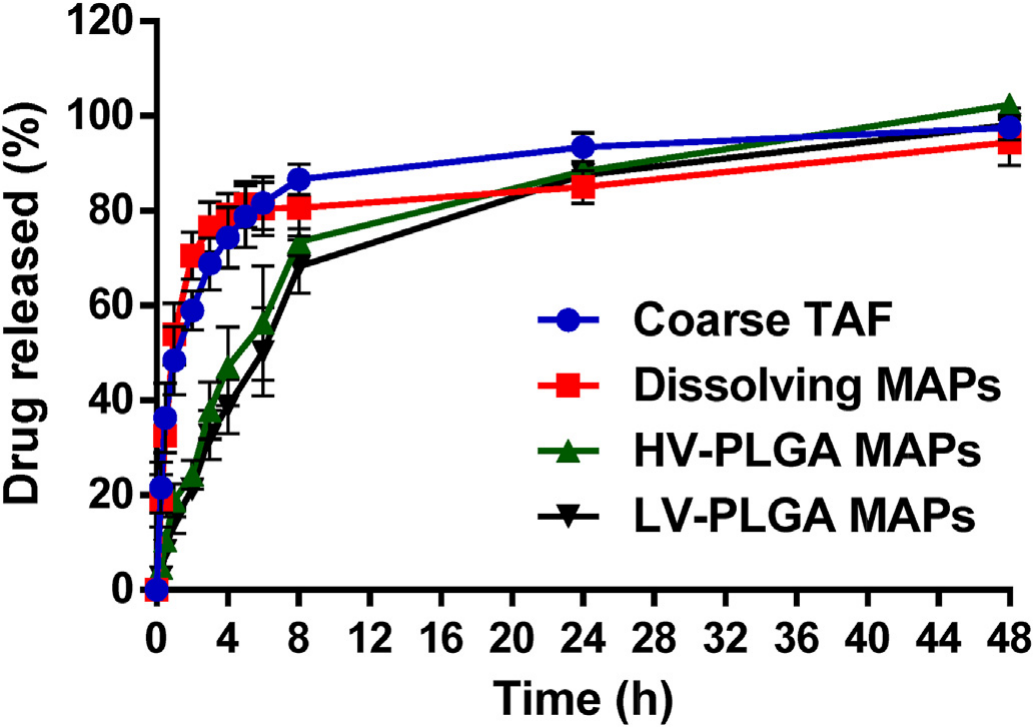
*In vitro* cumulative release profile obtained using a dialysis membrane model for the coarse drug (TAF powder), dissolving MAPs, and implantable MAPs. The MWCO of the dialysis membranes was 12,000–14,000. Release experiments were carried out in quadruplicate (*n ​=* ​4) and results were expressed as means ​± ​SD.

The release profiles obtained using the dialysis membrane model are presented in Fig. 7. This experiment evidenced a rapid release of the drug from the dissolving MAPs, with no significant differences observed between this group and the coarse drug (*p* ​> ​0.05). For example, at 4 ​h, the dissolving formulation and the coarse drug released 79.17 ​± ​4.43% and 74.34 ​± ​6.40%, respectively. When considering the LV- and HV-PLGA MAPs, both polymers presented a similar release behaviour, and no significant differences were found between both groups (*p* ​> ​0.05). For instance, at 4 ​h, the LV-PLGA formulation released 37.78 ​± ​5.71% of the drug, whereas the HV-PLGA 47.11 ​± ​8.30%. In comparison with the dissolving formulations, significant differences were only found until 6 ​h (*p* ​< ​0.05 in both cases), where the amount of TAF released for dissolving, LV-PLGA and HV-PLGA MAPs were 80.42 ​± ​5.61%, 56.31 ​± ​12.03% and 50.22 ​± ​9.29%, respectively. Nevertheless, after 6 ​h, the four samples assayed demonstrated similar amounts of drug released, reaching approximately 90% at 24 ​h and 95% at 48 ​h. TAF is a hydrophilic molecule and its solubility in water at room temperature is of 5.63 ​± ​0.54 ​mg/ml. At the sink conditions used in this study, the drug easily dissolved and diffused through the dialysis membrane to the bulk release media. PLGA is bioresorbable that has been widely used to manufacture drug delivery systems, including MAPs [38,41,44]. In the MAPs reported here, the drug escaped the PLGA matrix with relative rapidity, which can be explained by the high ratio between drug and polymer (1:1 w/w) in the final formulation, which led to a burst release of the drug when drug particles dissolved creating pores through which the release media permeated to further dissolve the drug contained in the MN matrix. Importantly, we aimed to achieve the highest possible amounts of drug loaded in the MAPs, which is critical when thinking of a final product with a discrete patch size and acceptable mechanical properties.

### 3.7 Skin deposition experiments

**Fig. 8:**
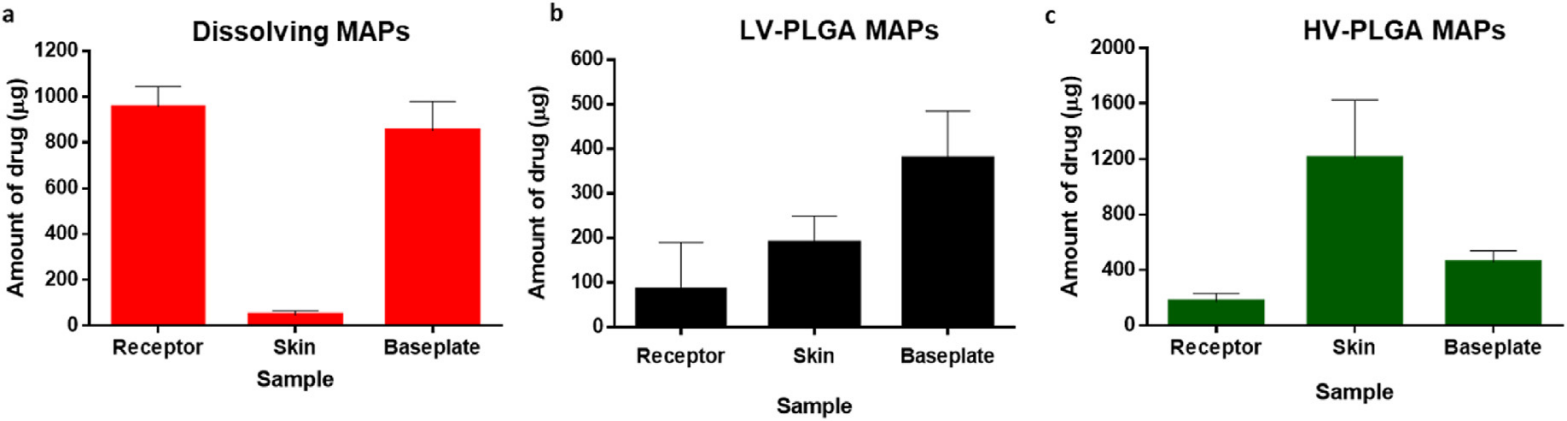
*Ex vivo* skin deposition studies in excised neonatal full-thickness porcine skin evaluated in Franz cells. Dissolving and PLGA MAPs were assayed by sextuplicate (*n ​=* ​6), and results expressed as means ​± ​SD.

*Ex vivo* skin deposition studies in Franz cells were performed in order to assess the ability of the novel MAPs to deliver TAF to neonatal full thickness porcine skin. As can be observed in Fig. 8, when dissolving MAPs were applied the amount of drug extracted from the skin after 24 ​h was 47.87 ​± ​16.33 ​μg, whereas a total amount of TAF released into the receptor compartment of the Franz cells was 957.12 ​± ​88.74 ​μg. Moreover, 853.3 ​μg of drug was found in the baseplates after they were removed from the skin, which was attributed to the hydrophilic characteristics of TAF and its resulting potential for back-migration. When the polymers that form the MN tips hydrate and dissolve, the drug dissolves too and back-migrates together with the fluids of the skin, dissolving the second layer of the MAPs made of high viscosity PVP K90, leading to the formation of a drug-containing residual gel that remains attached to the baseplates. PLGA MAPs were more efficient in keeping the active deposited in the skin. However, marked differences were observed between the two PLGA formulations. LV-PLGA MAPs were able to deposit 189.52 ​± ​60.23 ​μg of TAF in the skin and deliver nearly 90 ​μg to the receptor compartment, whereas the vast majority of the drug remained attached to the baseplates in the form of MNs that did not insert well in the skin due to the poor mechanical properties of this formulation, as discussed in section 3.2. HV-PLGA MAPs, on the other hand, showed the greatest ability to deliver TAF to the skin, depositing 1208.04 ​± ​417.9 ​μg of TAF, which represents values 25 and 6 times higher than those observed for dissolving and LV-PLGA MAPs, respectively. Moreover, the superior insertion capacity of this formulation led to improved MN insertion and lower amounts of TAF being quantified in the baseplates (457.84 ​± ​80.17 ​μg). Regardless of the formulation type, once the drug has been deposited in the viable layers of the skin, which are rich in fluids, it dissolves and diffuses to deeper layers of the dermis, eventually reaching the receptor compartment of the Franz cells. This explains why nearly 1 ​mg of the active ended up in the receptor compartment when using the dissolving formulations, which after the low molecular weight PVP and PVA dissolved, exposed the drug particles to dissolution and diffusion. The HV-PLGA formulation had an improved ability to retain the drug in the skin and release it in a slower rate than dissolving MAPs, which is in agreement with the lower amounts of TAF detected in the receptor compartments after 24 ​h (172.05 ​± ​59.05 ​μg). The delivery efficiencies of dissolving, LV-PLGA and HV-PLGA were 39.84%, 10.69% and 59.25%, respectively. Whereas the low drug delivery efficiency of LV-PLGA MAPs was related to the poor mechanical properties of the formulation, dissolving and HV-PLGA MAPs showed similar drug delivery efficiencies to hose observed previously in the literature using similar formulations [41,45,46].

### 3.8 Transdermal cumulative permeation

**Fig. 9:**
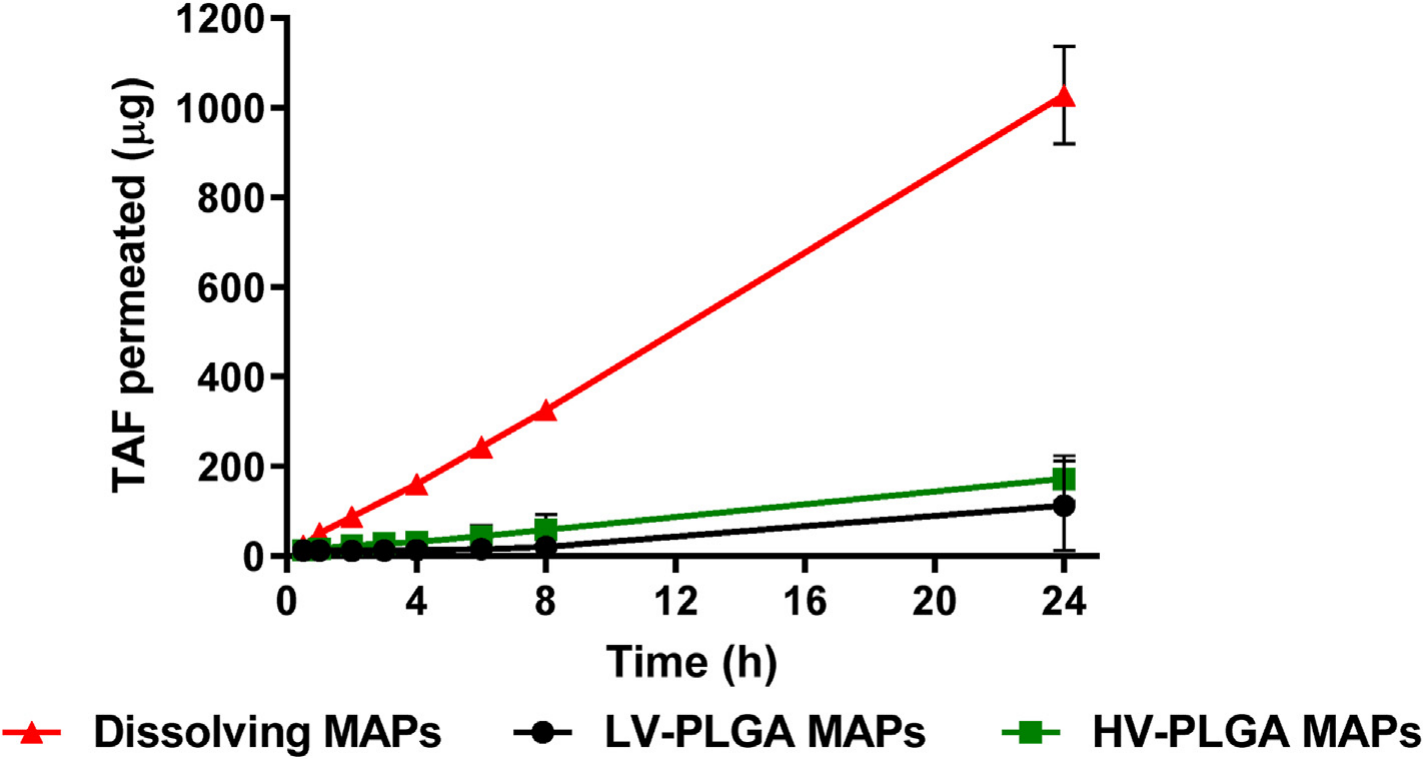
Transdermal cumulative permeation evaluated rate in Franz cells for dissolving and implantable LV- and HV-PLGA MAPs. Experiments were carried out by sextuplicate (*n* ​= ​6), and the results expressed as means ​± ​SD.

The amount of drug able to diffuse through the different layers of the skin to the receptor compartment can be used a good predictor of the *in vivo* pharmacokinetic behaviour of MAPs. Among the formulations reported here, dissolving MAPs were able to deliver the greatest amount of drug, with more than 1 ​mg of TAF reaching the receptor compartment after 24 ​h, an amount that was 6 times higher than that observed for PLGA MAPs (Fig. 9). The cumulative permeation profile was linear for all the MAPs, and PLGA formulations did not show any significant differences on the final amount of drug that reached the receptor compartment of the Franz cells. In contrast with the release data obtained from the dialysis membrane experiments, the transdermal cumulative permeation was slower, probably because of the reduced amount of fluids available for drug dissolution in the skin. Here, the dissolved active must diffuse through the different aqueous layers of the skin in order to get the dissolution media in the receptor compartment of the Franz cells. Other hydrophilic molecules in high doses have been delivered to the skin using microneedles based formulations, producing a rapid detection of the drug in the receptor compartment, which also corresponded with a high *in vivo* absorption [47,48]. Therefore, pharmacokinetics studies to the novel TAF MAPs developed here were required to prove their utility in the delivery of the drug into the systemic circulation.

### 3.9 Optical coherence tomography

**Fig. 10:**
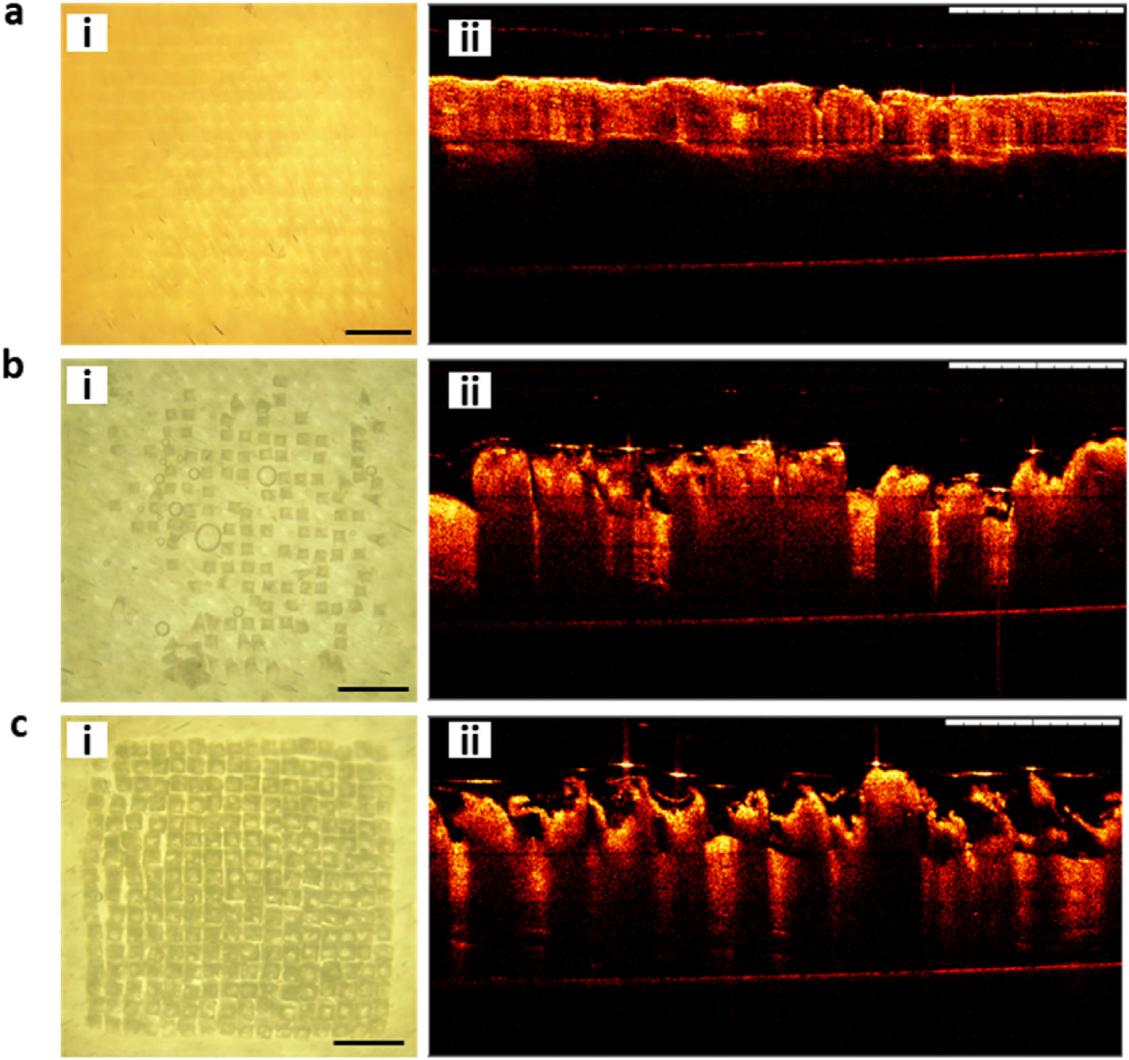
Insertion of MNs evaluated by optical microscopy and OCT 24 ​h post-application in a Franz cells experiment. **a-** Optical microscopy (i) and OCT (ii) images of dissolving MAPs, **b-** Optical microscopy (i) and OCT (ii) images of LV-PLGA MAPs, and c- Optical microscopy (i) and OCT (ii) images of HV-PLGA MAPs. Scale bar ​= ​1 ​mm.

The mechanical characterisation and skin deposition experiments showed, in a complementary manner, that dissolving and HV-PLGA MAPs were efficient in penetrating the skin and releasing TAF to the dermis with different rates. In order to confirm the physical insertion of the MNs in the skin, optical microscopy images and OCT were used. As can be seen in Fig. 10a, dissolving MAPs were able to pierce the skin, leaving a pattern of marks in the area where the MAP was applied, which were observed against the light in the optical microscopy analysis, and as small indentations in the OCT images. LV-PLGA MAPs presented a poor penetration capacity in the skin, with only a small amount of MNs inserted, as confirmed by the optical microscopy and OCT images (Fig. 10b). Finally, the HV-PLGA MAPs produced full insertion of the MNs as evidenced in the regularly spaced square structures observed in the optical microscopy (Fig. 10c). The OCT analysis of the HV-PLGA MAPs further confirmed that the MN tips remained in the skin after the 24 ​h experiment.

### 3.10 Pharmacokinetic experiment

**Fig. 11:**
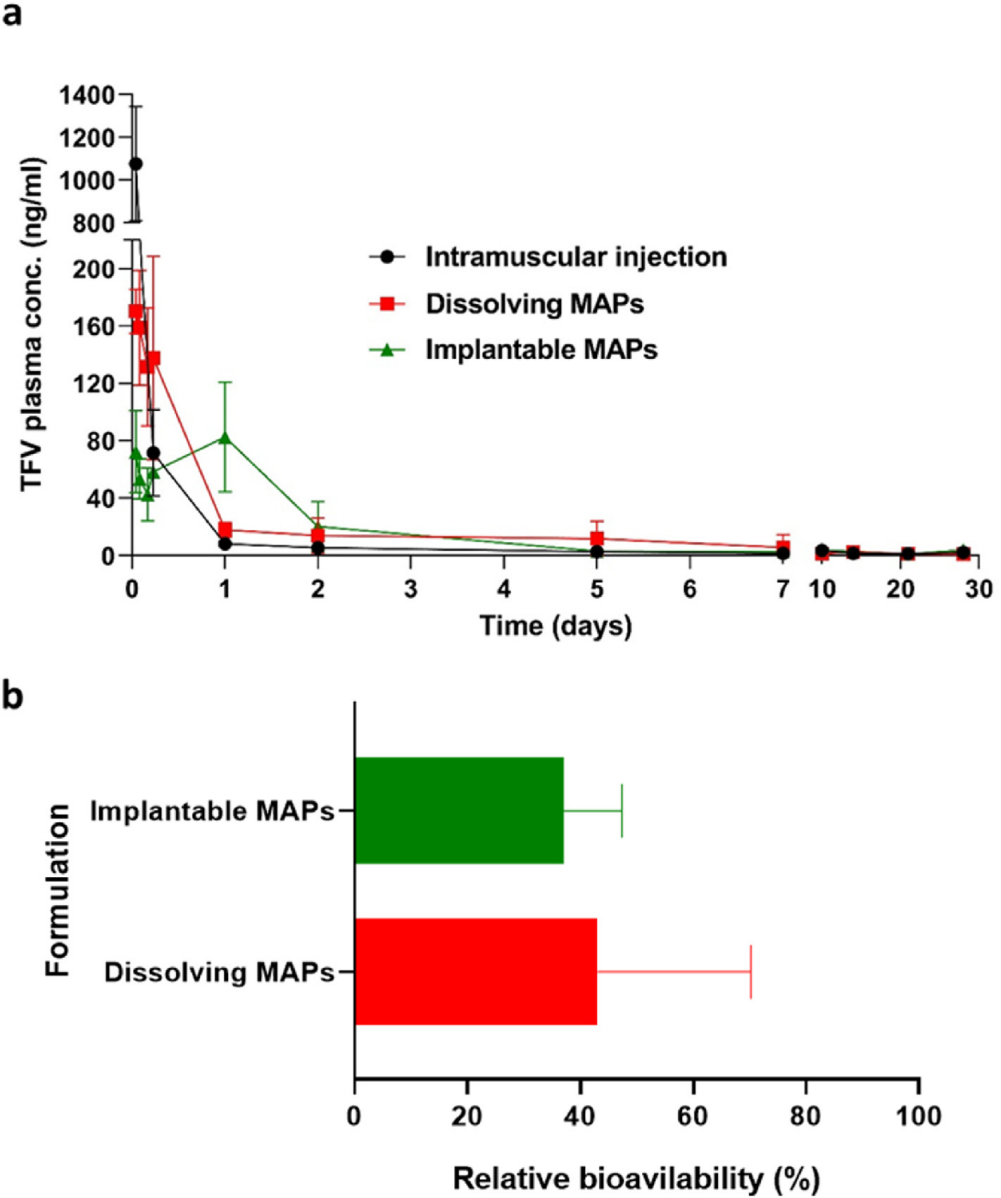
**a-** Plasma concentrations of the metabolite TFV obtained after the application of an IM injection of TAF, and TAF dissolving and PLGA MAPs to rats. **b-** Relative bioavailability of TAF after application of dissolving and PLGA MAPs to rats using an IM injection as a control. IM dose per animal: 4 ​mg, Dissolving MAPs dose per animal: 9.04 ​mg, implantable MAPs dose per animal: 9.48 ​mg. Experiments were carried out by sextuplicate (*n* ​= ​6), and the results expressed as means ​± ​SD.

The pharmacokinetic data obtained after an IM injection of TAF and the application of dissolving and HV-PLGA MAPs are shown in Fig. 11 and Table 3. The pharmacokinetic profiles indicate a rapid absorption and metabolism of TAF to TFV, since the detected presence of the parent drug in plasma was negligible. The IM injection resulted in an early peak in TFV plasma levels (904.79 ​± ​310.80 ​ng/ml) observed at 1 ​h (T_max_ ​= ​0.06 ​± ​0.02 ​d), followed by a rapid decrease in the plasma concentrations, which then stabilised at 24 ​h, leading to a plateau of approximately 8 ​ng/ml until day five, after which the plasma levels of TFV were below 3 ​ng/ml. Concentrations of TFV above the LLQ of the bioanalytical method were found in the plasma samples corresponding to the IM injection until day 14 of the study (1.12 ​± ​1.01 ​ng/ml), dissolving MAPs until day 7 (5.74 ​± ​8.78 ​ng/ml) and implantable MAPs until day 10 (2.39 ​± ​4.01 ​ng/ml).

**Table 3:** Pharmacokinetic data of the metabolite TFV in rat plasma after application of a TAF intramuscular injection, dissolving and PLGA TAF MAPs. Experiments were carried out by sextuplicate (*n* ​= ​6), and the results expressed as means ​± ​SD.

	Formulation		
Parameter	**Intramuscular**	**Dissolving MAPs**	**Implantable MAPs**
T_1/2_ (d)	9.47 ​± ​8.81	3.71 ​± ​0.93^a^	12.73 ​± ​18.01
T_max_ (d)	0.06 ​± ​0.02	0.09 ​± ​0.07	0.82 ​± ​0.41^a^^,^^b^
C_max_ (ng/ml)	904.79 ​± ​310.80	175.10 ​± ​34.80^a^	85.79 ​± ​33.50^a^^,^^b^
AUC_0-28_ (ng/ml∗d)	202.64 ​± ​50.38	197.59 ​± ​69.40	189.40 ​± ​66.41
AUC_0-inf_ (ng/ml∗d)	227.36 ​± ​62.15	207.48 ​± ​68.00	229.68 ​± ​86.49
MRT (d)	7.88 ​± ​4.82	4.73 ​± ​1.11	12.47 ​± ​14.62

a Statistically significant difference with the IM formulation.

b Statistically significant difference with Dissolving MAPs. MAPs, Microneedle array patches; T_1/2_, TFV half-life; AUC, Area under the curve; MRT, mean residence time.

The administration of MAPs led to pharmacokinetic profiles that started with a decreasing trend in TFV concentrations followed by significantly different plasmatic peaks (T_max_) at 0.09 ​± ​0.07 and 0.82 ​± ​0.41 days for dissolving and implantable MAPs, respectively. Moreover, significant differences were found between maximum plasma concentrations (C_max_) of dissolving and implantable MAPs (175.10 ​± ​34.80 and 85.79 ​± ​33.50 ​ng/ml, respectively). Moreover, both MAPs formulations presented significant differences in C_max_ and T_max_ when compared to the IM control. Regarding AUC values, no significant differences were observed between the three cohorts which, in all cases, presented AUC of approximately 200 ​ng/ml∗d. Furthermore, non-significant differences were found in T_1/2_ and MRT values of TFV when delivered from the control, dissolving and implantable MAPs. Although several pharmacokinetic parameters were not statistically different between the MAPs groups and the control, as shown in Fig. 11b, the relative bioavailability of dissolving and implantable MAPs compared to the IM injection was 43.11 ​± ​27.06% and 37.05 ​± ​10.29%, respectively. Therefore, it indicates that the bioavailabilities of TFV following the administration of dissolving and implantable MAPs were lower than that of the IM injections.

According to clinical data, strict adherence to PrEP therapies of at least 4 doses per week can decrease the risk of HIV transmission by 96%, whereas the prevention outcomes for users with lower adherence resulted in only 42% of viral transmission prevention [[49], [50], [51], [52]]. Therefore, the simplification of ARV regimes, including novel ways of delivering such drugs is actively sought out by researchers in industrial and academic laboratories [13,53]. Previous studies by our Group have demonstrated the ability of dissolving MAPs to deliver the hydrophobic ARV drugs rilpivirine, cabotegravir and etravirine systemically in rats in a long-acting manner [34,54,55].

To date, there is only limited knowledge regarding the administration of TAF through the skin. Jiang et al., for example, reported a transdermal patch that was able to release the drug for a week [28]. This study demonstrated that TAF was able to cross the *stratum corneum* and be absorbed into the systemic circulation in hairless rats. However, the authors recognised that the area of the patch should be multiplied at least several times to reach EC_50_ for anti-HIV activity in humans. Another report described the release of TAF from subdermal implants in dogs, achieving median plasma concentrations (40 days) of 0.85 ​ng/ml for TAF and 15 ​ng/ml for TFV. These values corresponded with efficient loading of peripheral blood monocyte cells (PBMC) with TFV diphosphate (TFV-DP) [23]. A refillable subdermal implant described by Chua et al. [25], was able to release TAF and TFV into the systemic circulation for 70 days in rhesus macaques, achieving median plasma concentrations of 0.1 ​ng/ml for TAF and 3.5 ​ng/ml for TFV, which again corresponded with therapeutically relevant intracellular concentrations of TFV-DP (40 fmol/10^6^ PBMC). A recent report from Pons-Faudoa et al., described the delivery of TAF fumarate from a nanofluidic implant in non-human primates for 4 months, maintaining median plasma concentrations of 0.51 and 7.81 ​ng/ml for TFV and TAF, respectively [27]. These values correlated with a TFV-DP concentration of 390 fmol/10^6^ PBMC. Crucially, the preventive efficacy of this implant against HIV infection in the same animal model was of 62.50% [27]. Considering the reports described above, implants seem to hold promise in keeping constant plasma concentrations of TAF. Nevertheless, the use of such systems in areas with underdeveloped healthcare systems still seems challenging.

Ruane et al. reported that a single oral dose of 25 ​mg/day for 10 days in HIV-1-infected people produced a TFV C_max_ of 15.7 ​ng/ml and the highest median decrease from log_10_c/ml HIV-1 RNA in comparison to a dose of 8 ​mg/day [56]. At day 7 of our study, the TFV levels were of 5.74 ​± ​8.78 ​ng/ml and 3.12 ​± ​3.80 ​ng/ml for dissolving and implantable MAPs. These plasma concentrations could hypothetically correspond to intracellular levels of TFV-DP above the therapeutic threshold. However, due to the low volumes of blood that can be obtained from rats, it is practically unfeasible to isolate PMBC for intracellular drug quantification. We showed in the present study that both microneedle types were capable of yielding physiologically-relevant TFV concentrations in rats and that these levels were maintained for around 7 days, being, after an initial injection-induced peak, similar to those achieved using a conventional intramuscular injection. Whereas other ARV drugs such as rilpivirine and cabotegravir produced sustained release for several weeks in rats when delivered using dissolving MAPs [34,54], TAF MAPs presented a limited capacity for long-acting drug delivery. This was attributed to the physicochemical and pharmacokinetic characteristics of the drug, i.e., its hydrophilic nature, rapid metabolization and a short half-life. This work shows that for TAF, changes at molecular level would need be done, such as, synthesis of a more hydrophobic salt, or the formation of a cocrystal with another more hydrophobic antiretroviral drug. Despite that, this proof-of-concept study proved that both the dissolving and implantable MAPs described could be a useful platform for systemic delivery of TAF.

A minimally invasive device that allows systemic distribution of ARV drugs is highly desirable in the treatment and prevention of HIV. Further developments on the patches described here could lead to a user-centric product that can be self-applied and provide protection for prolonged periods than a tablet, without the pain associated with IM injections. These MAPs could also avoid the creation of sharps waste and be distributed among communities living in rural areas without the need for refrigeration or aid from trained health-care personnel for administration.

## 4 Conclusions

Our work aimed to investigate, from a fundamental science viewpoint, the ability of two important types of microneedle patches to deliver TAF systemically. MAPs can play a key role in the development of novel regimens for HIV treatment and PrEP, replacing current oral and injectable formulations and leading to increased user compliance. This work reports for the first time the delivery of TAF to the systemic circulation using MAPs with dissolving and implantable MN tips. Both formulations were mechanically strong for excised neonatal porcine skin penetration and presented a rapid release of the drug *in vitro*, with PLGA showing only a limited capacity of controlling TAF release beyond the first 24 ​h. Skin experiments, on the other hand, revealed that the implantable PLGA MN tips were able to deposit considerable amounts of drug in the skin and release it transdermally. Finally, the pharmacokinetic experiments demonstrated a complete metabolization of TAF into TFV, with TFV being detected in plasma up to 7 days. This proof-of-concept study demonstrates the ability of both dissolving and implantable MAPs for the delivery of TAF. However, further experiments in bigger animal species, such as rhesus macaques, could allow us to understand in greater depth other translational aspects related to the MAPs described in this work.

## Credit author statement

Alejandro J. Paredes: Conceptualization, Methodology, Investigation, Formal analysis, Writing – original draft, Writing – review & editing. Fabiana Volpe-Zanutto: Methodology, Investigation. Lalitkumar K. Vora: Methodology, Investigation. Ismaiel A. Tekko: Methodology, Investigation. Andi Dian Permana: Methodology, Investigation. Camila J. Picco: Methodology, Investigation. Helen O. McCarthy: Writing – review & editing. Ryan F. Donnelly: Conceptualization, Supervision, Funding acquisition, Writing – original draft, Writing – review & editing.

## Declaration of competing interest

The authors declare that they have no known competing financial interests or personal relationships that could have appeared to influence the work reported in this paper.
